# Evidence for the occurrence of two sympatric sibling species within the *Anopheles (Kerteszia) cruzii* complex in southeast Brazil and the detection of asymmetric introgression between them using a multilocus analysis

**DOI:** 10.1186/1471-2148-13-207

**Published:** 2013-09-24

**Authors:** Luísa DP Rona, Carlos J Carvalho-Pinto, Alexandre A Peixoto

**Affiliations:** 1 Universidade Federal do Rio de Janeiro, Polo de Xerém, Estrada de Xerém 27, Duque de Caxias 25245-390, RJ, Brazil; 2 Departamento de Biologia Celular, Embriologia e Genética, CCB, Universidade Federal de Santa Catarina, Florianópolis 88040-970, SC, Brazil; 3 Departamento de Microbiologia e Parasitologia, CCB, Universidade Federal de Santa Catarina, Florianópolis 88040-970, SC, Brazil; 4 Laboratório de Biologia Molecular de Insetos, Instituto Oswaldo Cruz, FIOCRUZ, Av. Brasil 4365, Rio de Janeiro 21040-360, RJ, Brazil; 5 Instituto Nacional de Ciência e Tecnologia em Entomologia Molecular, Rio de Janeiro, RJ, Brazil

**Keywords:** *Anopheles*, Speciation, Complex of cryptic species, Multilocus analysis, Malaria, Mosquitoes

## Abstract

**Background:**

*Anopheles (Kerteszia) cruzii* (Diptera: Culicidae) is a primary vector of human and simian malaria parasites in southern and southeastern Brazil. Earlier studies using chromosome inversions, isoenzymes and a number of molecular markers have suggested that *An. cruzii* is a species complex.

**Results:**

In this study, a multilocus approach using six loci, three circadian clock genes and three encoding ribosomal proteins, was carried out to investigate in more detail the genetic differentiation between the *An. cruzii* populations from Florianópolis–Santa Catarina (southern Brazil) and Itatiaia–Rio de Janeiro States (southeastern Brazil). The analyses were performed first comparing Florianópolis and Itatiaia, and then comparing the two putative sympatric incipient species from Itatiaia (Itatiaia A and Itatiaia B). The analysis revealed high *F*_*ST*_ values between Florianópolis and Itatiaia (considering Itatiaia A and B together) and also between the sympatric Itatiaia A and Itatiaia B, irrespective of their function. Also, using the IM program, no strong indication of migration was found between Florianópolis and Itatiaia (considering Itatiaia A and B together) using all loci together, but between Itatiaia A and Itatiaia B, the results show evidence of migration only in the direction of Itatiaia B.

**Conclusions:**

The results of the multilocus analysis indicate that Florianópolis and Itatiaia represent different species of the *An. cruzii* complex that diverged around 0.6 *Mya*, and also that the Itatiaia sample is composed of two sympatric incipient species A and B, which diverged around 0.2 *Mya*. Asymmetric introgression was found between the latter two species despite strong divergence in some loci.

## Background

The *Anopheles* mosquitoes are insects belonging to order Diptera, family Culicidae and subfamily Anophelinae [1]. The genus *Anopheles* comprises almost 500 formally named species, although many of them are complexes of sibling species [2,3]. This genus is subdivided into six subgenera, among them *Kerteszia*[1,4]. *Anopheles* (*Kerteszia*) *cruzii* is a Neotropical mosquito highly specialized in using bromeliad plants as breeding reservoirs during the development of immature stages. This bromeliad-breeding mosquito is mainly found in Atlantic forest areas along the Brazilian coast, a habitat rich in plants from the Bromeliaceae family [4-6]. This mosquito is an important vector of human and simian malaria parasites in southern and southeastern Brazil [7,8], essentially in forest environments [9-11].

Different studies have been made for identification of *Anopheles* species complexes, which are virtually identical in adult morphology, but exhibit differences in their malaria transmission competence, resting habitats, host preference and insecticide resistance [3,12-14]. In this way, many authors have used molecular and genetic data for evolutionary analysis in a number of *Anopheles* species (*An. gambiae*, *An. fluviatilis*, *An. funestus*, *An. barbirostris*, *An. punctulatus*, *An. subpictus* and others) in an effort to resolve their taxonomic status [2,13,15-21]. Despite all accumulated molecular and genetic data originated from these studies, there are only a few population genetic studies of *An. cruzii* and most of them suggest that *An. cruzii* is a species complex. Differences in *X* chromosome inversions frequencies from populations of southeastern and southern Brazil, suggested the existence of three *An. cruzii* sibling species [22,23]. On the other hand, isoenzymes indicated two genetically isolated groups, one from Bahia State (northeastern Brazil), and the other from southeastern and southern Brazil (Rio de Janeiro, São Paulo and Santa Catarina States) [24]. These studies led to further analyses using different molecular markers. A fragment of the *timeless* gene, a locus involved in the control of circadian rhythms, was used as a marker to assess the genetic differentiation among a number of *An. cruzii* populations. The results indicated that a population of *An. cruzii* from northeast Brazil constitutes a highly differentiated group when compared with other five populations from the south and southeast regions of the country [25]. In addition, a multilocus approach using six loci, three circadian clock genes and three encoding ribosomal proteins, was implemented to investigate in more detail the genetic differentiation and time of divergence between the populations of the northeast and south Brazil. The analysis revealed very high *F*_*ST*_ values and fixed differences between these two cryptic species in all six loci, irrespective of their function. This analysis also indicated that they probably have not exchanged migrants since their separation, which was roughly estimated to have occurred around 2.4 million of years ago [26].

Besides, a fragment of the *cpr* gene, a locus involved in metabolic insecticide resistance and odorant clearance in insects, was used to analyze the divergence between *An. cruzii* populations from south and southeast Brazil (Florianópolis–SC, Cananéia and Juquitiba–SP, Itatiaia–RJ, Santa Teresa–ES). The *cpr* gene revealed very high *F*_*ST*_ values and fixed differences between Itatiaia and the other four populations studied. The data also provided preliminary evidence for the possible occurrence of two sympatric incipient sibling species in Itatiaia, called by the authors Itatiaia A and Itatiaia B [27].

In the current study, a multilocus analysis using six different nuclear gene fragments was performed comparing Florianópolis and Itatiaia populations. Additional multilocus analyses were also carried out considering Itatiaia as two different incipient species (Itatiaia A and Itatiaia B). The aim of the study was to determine if there is gene flow between the putative sibling species and to estimate their divergence time.

## Results

One of the assumptions of the Isolation with Migration model used in this study is the absence of recombination within the studied loci. In order to fulfill this requirement, the optimal recombination-filtered block was extracted from each gene alignment. Additional file 1: Table S1 shows the position of the non-recombining (NR) blocks used in this study as well as the putative recombinant sequences that were removed (see Methods). Another assumption of the IM program is that the variation observed in the studied loci is neutral. Therefore, the Tajima [28] and Fu & Li [29] tests of neutrality were used and the results are presented in Additional file 2: Table S2, together with the polymorphism summaries of the six gene fragments. No significant deviations from neutrality were observed in all comparisons. Additional file 2: Table S2 also shows the minimum number of recombination events for each gene (*RM*) and the length of the whole fragment and for the NR block of each gene (values in parentheses). The alignments of the whole sequences of each gene are presented in Additional file 3: Table S3. All loci include at least one intron of variable size, except the *cyc* gene fragment, which was composed entirely of an exon. Besides, Additional file 2: Table S2 shows the number of polymorphic sites (S) for each *An. cruzii* sibling species and two measures of nucleotide diversity: π, based on the average number of pairwise differences and θ, based on the total number of mutations (values for the NR blocks in parentheses). Pairwise estimates of population differentiation between the *An. cruzii* sibling species are shown in Table 1, which also shows the average number of nucleotide substitutions per site (*Dxy*), the number of net nucleotide substitutions per site between species (*Da*) and the distribution of the four mutually exclusive categories of segregating sites observed in each comparison: the number of exclusive polymorphisms for each species (*S*_*1*_ and *S*_*2*_), the number of shared polymorphisms (*S*_*s*_) and the number of fixed differences (*S*_*f*_).

**Table 1:** Genetic differentiation between populations

**Locus**	**Populations**	***F***_***ST***_	**P (*F***_***ST***_**)**	***Dxy***	***Da***	***S***_***s***_	***S***_***f***_	***S***_***1***_	***S***_***2***_
***Timeless***	***Florianópolis X Itatiaia***	0.1838 (0.2661)	0.000 (0.000)	0.03073 (0.02763)	0.00564 (0.00735)	19 (02)	00 (00)	44 (19)	11 (03)
	***Florianópolis X Itatiaia A***	0.3154 (0.2876)	0.000 (0.000)	0.03058 (0.02813)	0.01116 (0.00809)	07 (01)	02 (00)	43 (20)	03 (01)
	***Florianópolis X Itatiaia B***	0.1892 (0.3000)	0.000 (0.000)	0.02859 (0.02433)	0.00646 (0.00730)	09 (01)	00 (00)	40 (20)	09 (00)
	***Itatiaia A X Itatiaia B***	0.3418 (0.2081)	0.0000 (0.0300)	0.0223 (0.0120)	0.0076 (0.0025)	05 (01)	02 (00)	05 (04)	13 (03)
***Clock***	***Florianópolis X Itatiaia***	0.6707 (0.6340)	0.000 (0.000)	0.05159 (0.05930)	0.03460 (0.03760)	00 (00)	01 (01)	06 (06)	15 (14)
	***Florianópolis X Itatiaia A***	0.7325 (0.7207)	0.000 (0.000)	0.05065 (0.05879)	0.03710 (0.04237)	00 (00)	04 (04)	07 (07)	06 (05)
	***Florianópolis X Itatiaia B***	0.5735 (0.6926)	0.000 (0.000)	0.05463 (0.06270)	0.03133 (0.04343)	00 (00)	02 (04)	06 (07)	11 (04)
	***Itatiaia A X Itatiaia B***	0.1783 (0.0838)	0.0000 (0.1610)	0.0282 (0.0308)	0.0050 (0.0030)	03 (04)	00 (00)	03 (03)	14 (13)
***Cycle***	***Florianópolis X Itatiaia***	0.2845 (0.1765)	0.000 (0.000)	0.03575 (0.04093)	0.01017 (0.00723)	10 (06)	00 (00)	12 (07)	09 (02)
	***Florianópolis X Itatiaia A***	0.3510 (0.2236)	0.000 (0.000)	0.03600 (0.04284)	0.01264 (0.00958)	06 (04)	00 (00)	16 (09)	04 (02)
	***Florianópolis X Itatiaia B***	0.2127 (0.0863)	0.000 (0.029)	0.03278 (0.03550)	0.00697 (0.00307)	08 (04)	00 (00)	14 (09)	04 (00)
	***Itatiaia A X Itatiaia B***	0.1523 (0.1524)	0.0200 (0.1020)	0.0249 (0.0281)	0.0038 (0.0043)	05 (03)	00 (00)	05 (02)	07 (06)
***Rp49***	***Florianópolis X Itatiaia***	0.1667 (0.1303)	0.000 (0.000)	0.01433 (0.01155)	0.00239 (0.00151)	02 (01)	00 (00)	09 (09)	14 (11)
	***Florianópolis X Itatiaia A***	0.2985 (0.2228)	0.000 (0.000)	0.01147 (0.00860)	0.00342 (0.00192)	02 (01)	00 (00)	09 (09)	03 (02)
	***Florianópolis X Itatiaia B***	0.3743 (0.1978)	0.000 (0.000)	0.01774 (0.01453)	0.00664 (0.00288)	00 (00)	01 (00)	11 (10)	08 (07)
	***Itatiaia A X Itatiaia B***	0.4784 (0.4865)	0.0000 (0.0000)	0.0204 (0.0196)	0.0098 (0.0096)	00 (00)	01 (01)	05 (05)	08 (08)
***RpS2***	***Florianópolis X Itatiaia***	0.2631 (0.2647)	0.000 (0.000)	0.02045 (0.02005)	0.00538 (0.00531)	01 (00)	00 (00)	17 (15)	13 (11)
	***Florianópolis X Itatiaia A***	0.2961 (0.2539)	0.000 (0.000)	0.02066 (0.0199)	0.00612 (0.0050)	00 (00)	00 (00)	18 (15)	09 (08)
	***Florianópolis X Itatiaia B***	0.4212 (0.4880)	0.000 (0.000)	0.01924 (0.0201)	0.00811 (0.0098)	01 (00)	00 (00)	17 (15)	03 (03)
	***Itatiaia A X Itatiaia B***	0.4494 (0.4730)	0.0000 (0.000)	0.0153 (0.0152)	0.0069 (0.00723)	01 (01)	00 (00)	08 (07)	03 (02)
***RpS29***	***Florianópolis X Itatiaia***	0.7103 (0.7436)	0.000 (0.000)	0.05295 (0.07813)	0.03771 (0.05809)	01 (00)	05 (04)	12 (11)	24 (16)
	***Florianópolis X Itatiaia A***	0.7624 (0.7880)	0.000 (0.000)	0.0545 (0.0830)	0.0415 (0.0654)	01 (00)	05 (04)	13 (12)	10 (07)
	***Florianópolis X Itatiaia B***	0.7363 (0.7536)	0.000 (0.000)	0.0536 (0.0758)	0.0395 (0.0571)	01 (00)	04 (03)	13 (12)	14 (09)
	***Itatiaia A X Itatiaia B***	0.1819 (0.1675)	0.0000 (0.0110)	0.0216 (0.0208)	0.0039 (0.0035)	06 (02)	00 (00)	05 (03)	09 (10)

*F*_*ST*_, pair-wise estimates of population differentiation. *P*-value, significance of *F*_*ST*_ values (evaluated by 1,000 random permutations). *Dxy* and *Da*, average number of nucleotide substitutions per site and the number of net nucleotide substitutions per site between species, respectively [30]. *S*_*1*_, number of polymorphic sites exclusive to Florianópolis. *S*_*2*_, number of polymorphic sites exclusive to Itaparica. *S*_*s*_ and *S*_*f*_, number of shared polymorphisms and number of fixed differences between the two species, respectively. Numbers in parentheses are related to the non-recombining block (NR) for each locus.

The IM program was used to simultaneously estimate six demographic parameters (*θ*_*1*_, *θ*_*2*_, *θ*_*A*_, *t*, *m*_*1*_, *m*_*2*_) from the *An. cruzii* sibling species through an "Isolation with Migration" model using multiple loci [31]. As mentioned above, only the NR blocks were used and some recombining sequences were removed before the IM analysis (Additional file 1: Table S1). The analyses (see below) were performed first comparing Florianópolis and Itatiaia, then comparing the two putative sympatric incipient species from Itatiaia [27] and finally comparing those two putative siblings with Florianópolis. For each comparison, four independent runs were performed and among them, the simulations showed good convergence and consistency resulting in complete posterior distributions for all six parameters, except for *t* and *θ*_*A*_ in the simulations between Itatiaia A and Itatiaia B (see below).

Finally, gene trees of the sequences from all loci for both whole sequences (see below) and NR blocks (not shown) were estimated using the Neighbor-Joining method (NJ) with very similar results. The most suitable model selected using jModelTest 0.1.1 [32,33] was Kimura 2-parameter [34] for all loci. All trees were performed with 1,000 bootstrap replicates.

### Divergence between Florianópolis and Itatiaia

The polymorphism found in both populations was similar in most cases (Additional file 2: Table S2). The larger differences in length between the whole fragment and the NR block were observed for *tim* and *cyc* and this was due to the higher number of recombination events identified in these two genes (*RM* = 13 and 8 respectively). Except for the *tim* gene, all base substitutions were synonymous or occurred within introns (Additional file 2: Table S2). The few non-synonymous changes found in the *tim* fragment are described in Rona *et al*. [25].

Very high *F*_*ST*_ values were found using the *Clk* and *RpS29* genes between Florianópolis and Itatiaia (0.67 and 0.71, respectively) (Table 1). The other four genes showed *F*_*ST*_ values ranging from ~ 0.16 to 0.28. In all cases the *F*_*ST*_ values were significant. Florianópolis and Itatiaia had fixed differences and few or no shared polymorphisms in *Clk* and *RpS29* gene fragments. Among the other genes, there were shared polymorphisms and no fixed differences (Table 1).

**Figure 1:**
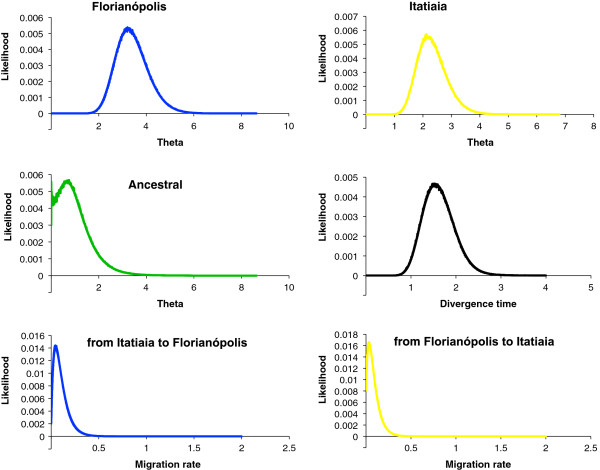
**Posterior probability distributions for each of the six demographic parameters estimated using IM for the pairwise comparison Florianópolis *vs *Itatiaia (A and B).** Effective population size for an ancestral and two descendent populations (*theta*), divergence time between Florianópolis and Itatiaia, and migration rates in both directions. Four IM simulations using different seed numbers were plotted for each parameter estimate (see also Additional file 4: Table S4). All curves are shown including the range of the priors.

Figure 1 shows the posterior probability distributions for each of the six demographic parameters estimated using IM and Additional file 4: Table S4 summarizes the features from the marginal histograms for each of the parameters in all MCMC runs. The estimates of *θ* suggest that the effective population size of the ancestral population was smaller than the current Florianópolis and Itatiaia populations indicating that both may have had a history of growth after separation (Figure 1).

Migration parameters have been estimated for all loci together for each population in different IM runs. Our aim was to detect the occurrence of gene flow using the multilocus data. The results have revealed no strong indication of migration in either direction using all loci together.

The divergence time parameter was estimated for all combined loci in four different IM runs (Table 2). This parameter cannot be directly converted to years because the mutation rates in *An. cruzii* species are unknown. Therefore, an estimate of the divergence time between *An. cruzii* species was performed using the average of *Drosophila* synonymous and non-synonymous substitution rates for several nuclear genes (0.0156 and 0.00191 per site per million year respectively) [35]. Using this approach and based on the average of HiSmth values, an estimate of the divergence time between Florianópolis and Itatiaia would be approximately 0.75 *Mya* (range from 0.51 to 1.1 *Mya*, based on the average of HPD90Lo and HPD90Hi values).

**Table 2:** **Divergence time estimates among the *An. cruzii *populations obtained by IM (based on the average of HiSmth values) and also by the average *Da *values from the six loci (based on the whole sequence)**

**Populations**	**IM**	***Da***
Florianópolis *x* Itatiaia (A and B)	0.75 *Mya*	0.51 *Mya*
Florianópolis *x* Itatiaia A	0.70 *Mya*	0.59 *Mya*
Florianópolis *x* Itatiaia B	0.80 *Mya*	0.52 *Mya*
Itatiaia A *x* Itatiaia B	-	0.19 *Mya*

Since the time parameter did not show good convergence in the IM runs between Itatiaia A and Itatiaia B, this divergence time was estimated using only the *Da* values.

Another method of estimating the divergence time between these two putative species is to use the same *Drosophila* synonymous substitution rate mentioned above and the average *Da* values from the six loci (Table 1). Based on these values, the divergence time between the populations from Florianópolis and Itatiaia was estimated to be approximately 0.51 *Mya* and 0.62 *Mya* for the whole sequence and NR blocks, respectively (Table 2).

**Figure 2:**
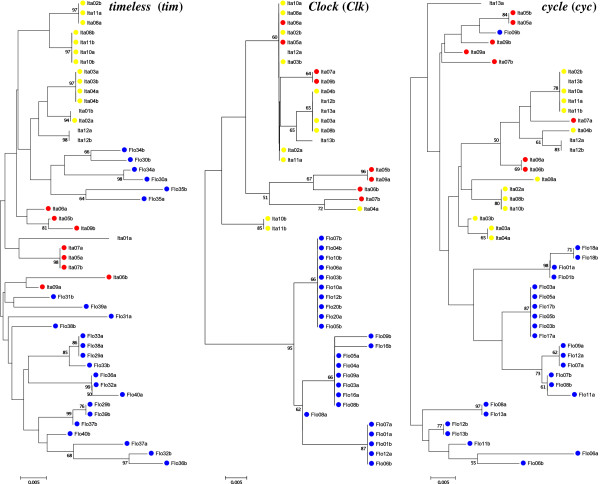
**Neighbor-joining trees of clock genes nucleotide sequences of Florianópolis and Itatiaia (A and B).** Neighbor-joining trees using the three clock genes nucleotide sequences of Florianópolis and Itatiaia (A and B) obtained with Kimura 2-parameter distance for all genes. Numbers on the nodes represent the percentage bootstrap values based on 1,000 replications. Flo: Florianópolis; Ita: Itatiaia; Itatiaia A: yellow circles; Itatiaia B: red circles.

**Figure 3:**
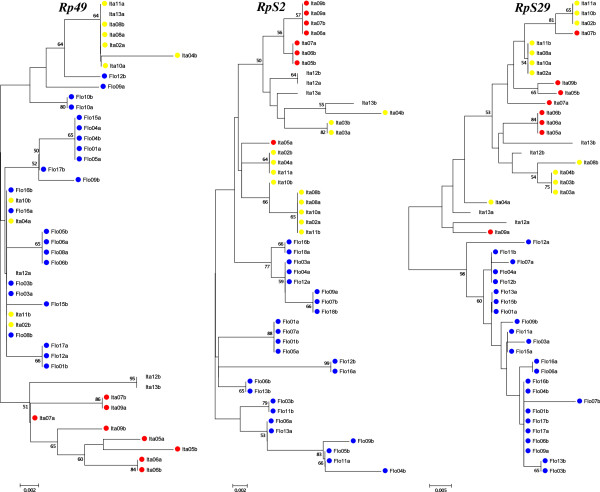
**Neighbor-joining trees of ribosomal protein genes nucleotide sequences of Florianópolis and Itatiaia (A and B).** Neighbor-joining trees using the three ribosomal protein genes nucleotide sequences of Florianópolis and Itatiaia (A and B) obtained with Kimura 2-parameter distance for all genes. Numbers on the nodes represent the percentage bootstrap values based on 1,000 replications. Flo: Florianópolis; Ita: Itatiaia; Itatiaia A: yellow circles; Itatiaia B: red circles.

The resulting NJ trees using *Clk* and *RpS29* clearly grouped the sequences from the two sibling species in different clusters with high bootstrap values (Figures 2 and 3).

### Divergence between Itatiaia A and Itatiaia B

Rona *et al*. [27] hypothesized that the Itatiaia sample might include two different sets of individuals based on *cpr* sequences. According to this classification the individuals Ita02, Ita03, Ita04, Ita08, Ita10 and Ita11 belong to Itatiaia A and the mosquitoes Ita05, Ita06, Ita07 and Ita09 belong to Itatiaia B. We therefore used our multilocus data to test the hypothesis that these two Itatiaia groups represent different sympatric incipient species. According to the *cpr* data, mosquito Ita12 was the only potential "hybrid" while Ita01 and Ita13 were not typed with this gene [27]. Therefore these three mosquitoes were not used in the analysis described below.

Itatiaia A was the least polymorphic, showing the lowest values of θ and π, as well as the smaller number of polymorphic sites (S) in most cases (Additional file 2: Table S2). High and significant *F*_*ST*_ values were observed between the two sympatric species, ranging from 0.15 to 0.47 (Table 1). In all cases the *F*_*ST*_ values were significant (except using the NR block of the *Clk* and *cyc* genes).

Itatiaia A and Itatiaia B had fixed differences and few or inexistent shared polymorphisms in *Rp49* and *RpS2* gene fragments, which show the highest values of differentiation (*F*_*ST*_ = 0.47 and 0.44, respectively). The *tim* locus also shown a high *F*_*ST*_ value (*F*_*ST*_ = 0.34) between these two putative species and fixed differences as well, but shared polymorphisms were also observed in this gene. Among the other loci, there were shared polymorphisms and no fixed differences (Table 1).

**Figure 4:**
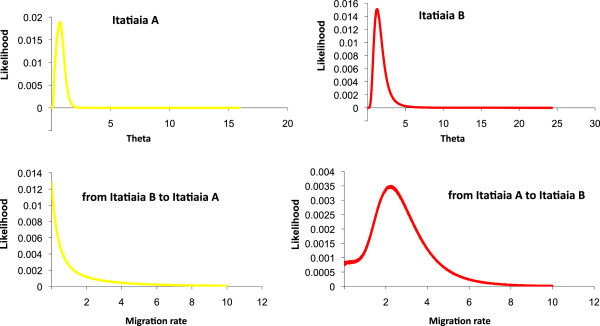
**Posterior probability distributions for each of the four demographic parameters estimated using IM for the pairwise comparison Itatiaia A *vs *Itatiaia B.** Effective population size for Itatiaia A and Itatiaia B (*theta*) and migration rates in both directions. Four IM simulations using different seed numbers were plotted for each parameter estimate (see also Additional file 5: Table S5). All curves are shown including the range of the priors.

Figure 4 shows the posterior probability distributions for each of the six demographic parameters estimated using IM and Additional file 5: Table S5 summarizes the features from the marginal histograms for each of the parameters in all MCMC runs for Itatiaia A and Itatiaia B. Migration parameters have been estimated for all loci together for each population in different IM runs. The results show evidence of migration only in the direction of Itatiaia B.

Since the *time* parameter did not show good convergence in the IM runs, the divergence time was estimated between Itatiaia A and Itatiaia B using only the *Drosophila* synonymous substitution rate and the average *Da* values from the six loci (Table 1) mentioned above. Based on these values, the divergence time between the populations from Itatiaia A and Itatiaia B was estimated to be approximately 0.19 *Mya* and 0.16 *Mya* for the whole sequence and NR blocks, respectively (Table 2).

The NJ trees (Figures 2 and 3) grouped the sequences from the two sibling species in different clusters using *tim*, *RpS2* and *Rp49* genes, which show the highest values of differentiation (*F*_*ST*_ = 0.34, 0.44 and 0.47, respectively). In the *RpS2* tree, there is a haplotype (Ita05a) from Itatiaia B among the Itatiaia A sequences. Also, the individuals Ita12 (the only potential "hybrid" typed with *cpr* gene) and Ita13 (that was not typed with *cpr*) appeared among Itatiaia A sequences. On the other hand, in the *Rp49* tree, these two mosquitoes showed a hybrid aspect, in agreement with *cpr* gene results, where Ita12 was the only potential "hybrid" [13]. The others, *Clk*, *cyc* and *RpS29*, did not show an evident separation in NJ trees between Itatiaia A and Itatiaia B.

### Divergence between Florianópolis *vs* Itatiaia A and Florianópolis *vs* Itatiaia B

In addition to the analyses performed between Florianópolis *vs* Itatiaia (considering Itatiaia A and B together) described above, analyses were also carried out comparing Florianópolis with Itatiaia A and Itatiaia B, separately.

The pairwise estimates of population differentiation between the two groups (Table 1) shown very high *F*_*ST*_ values using the *Clk* and *RpS29* genes, in agreement with the values found between Florianópolis and Itatiaia (considering Itatiaia A and B together). The further four genes shown *F*_*ST*_ values ranging from ~ 0.18 to 0.42. In all cases the *F*_*ST*_ values were significant. Interestingly, the clock genes (*tim, cyc* and *Clk* loci) shown the higher *F*_*ST*_ values between Florianópolis and Itatiaia A, whereas the *Rp49* and *RpS2* loci shown the higher *F*_*ST*_ values between Florianópolis x Itatiaia B. The *RpS29* showed very high and similar values in both comparisons (*F*_*ST*_ = 0.73 and 0.76).

Florianópolis and Itatiaia A had fixed differences and few or no shared polymorphisms in *Clk*, *RpS29* and *tim* gene fragments. Between Florianópolis and Itatiaia B, the loci *Clk, RpS29* and *Rp49* shown fixed differences and few or inexistent shared polymorphisms (Table 1).

**Figure 5:**
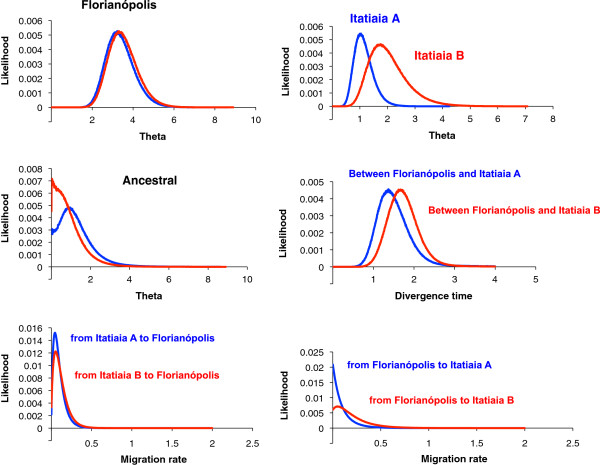
**Posterior probability distributions for each of the six demographic parameters estimated using IM for the pairwise comparisons Florianópolis *vs *Itatiaia A and Florianópolis *vs *Itatiaia B.** Effective population size for an ancestral and two descendent populations (*theta*), divergence time between Florianópolis and Itatiaia, and migration rates in both directions. Four IM simulations using different seed numbers were plotted for each parameter estimate (see also Additional file 6: Table S6). All curves are shown including the range of the priors.

Additional file 6: Table S6 summarizes the features from the marginal histograms for each of the parameters in all MCMC runs between Florianópolis *vs* Itatiaia A and between Florianópolis *vs* Itatiaia B, respectively, and Figure 5 shows the posterior probability distributions for each of the six demographic parameters estimated using IM for both groups. The estimates of *θ* suggest that Itatiaia A and Itatiaia B are smaller than Florianópolis population (Figure 5). Migration parameters have been estimated for all loci together for each population in different IM runs. The results have revealed no indication of migration in either direction using all loci together in both comparisons (Florianópolis *vs* Itatiaia A and Florianópolis *vs* Itatiaia B).

The divergence time parameter was estimated for all combined loci in four different IM runs. The IM runs indicated similar results between Florianópolis *vs* Itatiaia A, Florianópolis *vs* Itatiaia B and Florianópolis *vs* Itatiaia (considering A and B together), which suggest that the Itatiaia lineage separated from Florianópolis before splitting into A and B (Table 2). Based on the average of HiSmth values, an estimate of the divergence time between Florianópolis and Itatiaia A would be approximately 0.7 *Mya* and between Florianópolis and Itatiaia B would be approximately 0.8 *Mya*. Based on *Da* values, the divergence time between Florianópolis and Itatiaia A and B was estimated to be approximately 0.59 *Mya* and 0.52 *Mya*, respectively (Table 2).

## Discussion

The multilocus results presented here revealed a high level of differentiation between Florianópolis, Itatiaia A and Itatiaia B indicating that these populations represent indeed different species in the *An. cruzii* complex. Species as evolutionary lineages are expected to show greater evolutionary independence from one another than are populations within species [36]. Hey & Pinho [36] discuss the use of two measures of evolutionary independence, gene flow and divergence time, for species diagnosis, which help to differentiate intraspecific differences from interspecific differences. The two measures of evolutionary independence were also correlated with *F*_*ST*_ estimates. For simplicity, the authors suggested a threshold criterion of gene flow < 1, the separation time >1 (based on IM results) and the *F*_*ST*_ > 0.35 to species diagnosis. Among the six *F*_*ST*_ values calculated for the pairwise Florianópolis and Itatiaia (including A and B), only two of them are higher than 0.35 (*F*_*ST*_ = 0.67 and 0.71). Nevertheless, the mean *F*_*ST*_ among the six loci (0.38) and both measures of evolutionary independence (gene flow and divergence time) are above the threshold criteria. The comparison made between Itatiaia A and Itatiaia B showed similar results, but the migration parameter in the direction of Itatiaia B shown values higher than the suggested threshold, consistent with the idea of incipient speciation.

Previously, we observed no evidence of association between function (circadian x ribosomal genes) in the divergence levels between the sibling species of Florianópolis and Itaparica [26]. In the current work, *Clk* and *RpS29* showed the highest *F*_*ST*_ values between Florianópolis and Itatiaia (considering Itatiaia A and B together), while *Rp49* and *RpS2* showed the highest *F*_*ST*_ values between Itatiaia A and B. Interestingly, these four genes are located on the chromosome 2 of *Anopheles gambiae*, albeit the three ribosomal protein genes are on the right arm while *Clk* is on the left arm. Many authors have postulated that chromosomal inversions could promote speciation since their recombination suppressing effects facilitate the maintenance and accumulation of differences between interbreeding populations [37-39]. Ramirez & Dessem [22,23], studying the polytene chromosomes of *Anopheles cruzii* from south and southeast regions of Brazil, revealed the existence of three putative species, which differed mainly in the banding patterns of the *X* chromosome. Nevertheless, different autosomal inversions seemed to be associated with each form of *X* chromosome and it is tempting to speculate that some of the markers we used and showed high levels of differentiation between the sibling species are located within or nearby the inversions studied by Ramirez & Dessem [22,23]. Future *in situ* hybridization experiments using these markers as probes might confirm if that is the case.

The observation of a putative hybrid [27] and the varying degrees of differentiation observed in the six loci between Itatiaia A and Itatiaia B, suggest that these two siblings are in a process of incipient speciation. Similar results were found in the *Anopheles gambiae* complex [40-42] that maintains a genome in a mosaic form, with regions of low and high divergence. These variations among the different regions of the genome in the divergence between the *An. cruzii* siblings have two main explanations: i) the maintenance of ancestral polymorphisms as these cryptic species have separated recently and ii) differential introgression in different genomic regions between the species reflecting locations where gene flow occurs freely or is restricted by selection.

The two conflicting but no mutually exclusive hypotheses of retention of ancestral polymorphism and introgression between closely related species are often difficult to distinguished. Introgression can sometimes be excluded based on the geographic separation of two species and in this case, the shared polymorphisms can be classified as ancestral [43]. This is perhaps the situation found between the allopatric Florianópolis and Itatiaia (A and B) with no strong indication of migration, in either direction, as suggested by the IM results. On the other hand, the introgression hypothesis is of course more likely if the species are sympatric. This is probably the case of Itatiaia A and Itatiaia B, where the introgression hypothesis seem confirmed by the IM results that revealed nonzero values of migration parameter between the latter species, only in the direction of Itatiaia B.

Asymmetric introgression has been documented in a variety of taxa [44-48], including mosquitoes. For example, Donnelly *et al*. [43], comparing haplotype frequencies in allopatric and sympatric populations of *An. arabiensis* and *An. gambiae*, found unidirectional introgression, from the former into the latter species, only in sympatric populations. Gomes *et al*. [49] found differential introgression in the *Culex pipiens* complex, in spite of the high levels of genetic differentiation between its forms.

Marsden *et al*. [42] found similar results in the *An. gambiae* complex, between M and S molecular forms, and they argue, as one of the possible explanations, that the asymmetric introgression may be a consequence of the differences in relative abundance of the two taxa (S form–the genetic recipient–was more common than M form–the genetic donor–in all sites assessed), which could result in higher levels of backcrossing between the hybrids and the more abundant species. Our results are also consistent with this explanation as the estimates of population sizes and migration parameters from the IM program suggest that Itatiaia A (the genetic donor) has a smaller estimated population size than Itatiaia B (the genetic recipient).

Marsden *et al*. [42] also proposed that the asymmetric introgression can contribute to the maintenance of differentiation between M and S, since the unidirectional movement of nuclear genes from the M into the S form would prevent homogenization of their gene pools because of the conservation of unique polymorphism within the S form and a lack of admixture in M. Indeed, the observed genetic diversity was considerably lower in Itatiaia A than in Itatiaia B.

The estimated divergence time (based on the *Da* values) between Itatiaia A and Itatiaia B is ~ 0.2 *Mya* and between Florianópolis and Itatiaia (including A and B) is ~ 0.6 *Mya*. This indicates an earlier speciation process splitting the populations of Florianópolis and Itatiaia, followed by a more recent separation between the two sympatric species in the latter locality.

The time of divergence of these three sibling species as well as that between the Bahia and the south and southeast populations (~ 2.4 *Mya*) [26], points to the importance of Pleistocene environmental changes (glaciations and inter-glaciations periods) [50] as factors in the diversification of *An. cruzii* species complex. Since temperature and water are essential for the development of *Anopheles* spp. immature stages [4], the hypothesis that Pleistocene environmental changes driving *Anopheles* diversification seems plausible. Furthermore, in the case of *An. cruzii* (*Kerteszia* subgenus), its larval development is associated with water trapped in Bromeliads plants, which are restricted to the rainforest [4,7]. So, a number of studies show that several malaria vectors, including *An. cruzii*, have revealed patterns of genetic divergence associated with the Pleistocene environmental changes [26,51-55].

The process of successive rainforest contractions and expansions was a very important consequence of these environmental modifications possibly favoring the differentiation and speciation of forest obligate species [56-58]. Accordingly, a number of studies of closely related species demonstrated the importance of the Pleistocene climatic changes in shaping the Brazilian Atlantic forest biodiversity [59,60], since patterns of endemicity in many forest obligate species are concordant in geographic distribution [59,61-63]. For example, Mata *et al*. [64], studying the molecular phylogeny and biogeography of the eastern Tapaculo birds in Brazilian Atlantic forest, corroborated the importance of the Bahia refuge as an avian center of endemism and also concluded that the southeast (Serra da Mantiqueira, where Itatiaia is located) lineage is genetically different from the southern populations.

Turchetto-Zolet *et al*. [65] studying the molecular variation patterns in *Schizolobium parahyba* (Fabaceae), a widespread tree in the Brazilian Atlantic forest, found high levels of genetic diversity in populations from the southeast region and low levels in southern populations. They argue that the low genetic diversity in the south may be the result of a founder effect followed by a range expansion after glacial periods. Lorenz-Lemke *et al*. [66] and Palma-Silva *et al*. [67] also reported expansion toward the south in Atlantic forest plants. However, in the case of *An. cruzii*, Florianópolis (south sibling species) showed a higher level of genetic diversity than the Itatiaia (southeast) sympatric sibling species. Therefore, it is likely that there was a persistence of *An. cruzii* populations in the south during the contraction of the forest, as was proposed for toads [60], instead of a southern colonization of the Atlantic forest from northern regions.

## Conclusions

In summary, the results obtained with the studies of the *An. cruzii* complex suggest so far three main events of cladogenesis originating the different sibling species. The first one originated the Bahia (Itaparica) species and occurred toward the end of the Pliocene (~2.4 *Mya*) [25,26], the second that occurred around 0.6 *Mya* probably separated the Itatiaia lineage from other south and southeastern populations [25,27], and finally the most recent event (around 0.2 *Mya*) originated the new sympatric incipient species found in this locality. Analysis of a number of other populations of *An. cruzii* from Rio de Janeiro State, where Itatiaia is located, might allow us to determine the distribution of these two new sibling species and to investigated whether they might have possibly originated by a process of sympatric or parapatric speciation.

## Methods

### Molecular analysis

The mosquitoes used in this study were females captured in Florianópolis, Santa Catarina State (SC) (27°31′S / 48°30′W) and Itatiaia, Rio de Janeiro State (RJ) (22°27′S / 44°36′W). They were identified on the basis of their morphology according to Consoli and Lourenço-de-Oliveira [4]. For the molecular analysis, 12 individuals from Florianópolis and 10 to 12 from Itatiaia were used for each of the six gene fragments analyzed. Three of the fragments used are orthologues of *Drosophila melanogaster* genes involved in the control of circadian rhythms: *timeless* (*tim*), *Clock* (*Clk*) and *cycle* (*cyc*); and three code for ribosomal proteins: *Rp49* (Ribosomal protein 49, known also as *RpL32*–Ribosomal protein L32), *RpS2* (Ribosomal protein S2) and *RpS29* (Ribosomal protein S29).

The sequences of the six genes from Florianópolis and the sequences of the *tim* gene from Itatiaia were those previously published by our group [25,26] (Accession numbers: GU016330–GU016569 and FJ408732–FJ408865, respectively). The sequences of the other five genes from Itatiaia were obtained by PCR, cloning and sequencing as described below.

The primers used in this study were those previously published by our group [25,26]. The *An. cruzii* genomic DNA extracted according to Jowett [68] was used in PCR reactions carried out in an Eppendorf Mastercycler® thermocycler using the proofreading *Pfu* DNA polymerase (Biotools).

PCR products were purified and cloned using either Zero Blunt TOPO PCR cloning kit (Invitrogen) or pMOS Blue vector blunt-ended cloning kit (GE Healthcare). Sequencing of positive clones was carried out in an ABI Prism 3730 DNA sequencer at the Oswaldo Cruz Institute using the ABI Prism Big Dye Terminator Cycle Sequencing Ready Reaction kit (Applied Biosystems). The identity of the cloned fragments was determined by BlastX analysis using GenBank database [69].

At least eight clones were sequenced for each mosquito. Sequences were edited and in most cases consensus sequences representing the two alleles were generated. In a number of individuals only one haplotype was observed among the eight sequences and in these cases mosquitoes were classified as homozygotes. The probability of incorrectly classifying a heterozygote as a homozygote individual with this procedure is less than 1%. The sequences from homozygote mosquitoes were duplicated prior to analysis. However, when carried out without duplicating homozygote sequences, the analysis rendered similar results. Sequences were submitted to GenBank (Accession numbers: JX129234–JX129351).

### DNA sequence analysis

The sequences were aligned using ClustalX software [70] and population genetics analysis was carried out using DNASP4.0 [71] and P_RO_S_EQ_ v 2.91 [72] softwares.

The jModelTest version 0.1.1 [32,33] was used to find the most suitable model for each gene evolution. Models selected by the Bayesian Information Criterion (BIC) were favored and used in the phylogenetic analysis, carried out using MEGA 4.0 [73].

The IM program is an implementation of the Isolation with Migration model and is based on the MCMC (Markov Chain Monte Carlo) simulations of genealogies [31,74]. It simultaneously estimates six demographic parameters from multilocus data: effective population size for an ancestral and two descendent populations (*θ*_A_, *θ*_1_, and *θ*_2_, respectively), divergence time (*t*) and migration parameters in both directions (*m*_*1*_ and *m*_*2*_). Initial IM runs were performed in order to establish appropriate upper limits for the priors of each demographic parameter. These preliminary simulations generated marginal distributions that facilitated the choice of parameter values used in the final IM analyses. The convergence was assessed through multiple long runs (four independent MCMC runs with different seed numbers were carried out with at least 30,000,000 recorded steps after a burn-in of 100,000 steps) and by monitoring the ESS values, the update acceptance rates and the trend lines.

The Infinite Sites model [75] was chosen as the mutation model in the IM simulations because the two species are closely related and all genes are nuclear.

The optimal recombination-filtered block was extracted from each gene alignment using the IM_GC_ program, which also removes haplotypes that represent likely recombinant sequences [76,77].

## Competing interests

The authors declare that they have no competing interests.

## Authors’ contributions

LDPR participated in data generation and analysis, and drafted the manuscript. She also helped capture mosquitoes in Florianópolis. CJCP carried out the capture and morphological identification of mosquitoes. AAP is the principal investigator, participated in its design and coordination, and helped to write the manuscript. All authors read and approved the final manuscript.

## Supplementary Material

Additional file 1: Table S1 NR blocks and sequences excluded from the IM analysis. Edition of sequences prior to IM analysis using the IM_GC_ program and based on alignments presented in Additional file 3: Table S3. NR blocks, fragment positions of the non-recombining blocks used in the analyses; Removed sequences, the putative recombinant sequences removed before the IM analysis. Click here for file

Additional file 2: Table S2 Polymorphism summaries of *An. cruzii* sibling species. RM, the minimum number of recombination events; n, number of DNA sequences of each sibling species; S, number of polymorphic (segregating) sites; θ, nucleotide diversity based on the total number of mutations (Eta); π, nucleotide diversity based on the average number of pair-wise differences; *D*_*T*_, Tajima’s D [28]; *D*_*FL*_, Fu & Li’s D [29] and *F*_*FL*_, Fu & Li’s F [29], based on Eta (total number of mutations). No significant deviations from neutrality were observed after Bonferroni correction. Numbers in parentheses are related to the non-recombining block (NR) for each locus. Click here for file

Additional file 3: Table S3 Alignments of the DNA sequences of the *timeless, Clock, cycle, Rp49, RpS2 and RpS29* gene fragments from Florianópolis and Itatiaia. The translated amino acid sequences are shown above the alignments and the introns are highlighted in grey. Dots represent identity and dashed represent gaps. Flo: individuals from Florianópolis; Ita: individuals from Itatiaia. Click here for file

Additional file 4: Table S4 Summarized features of the marginal histograms for each parameter for the pairwise comparison Florianópolis *vs* Itatiaia. Click here for file

Additional file 5: Table S5 Summarized features of the marginal histograms for each parameter for the pairwise comparison Itatiaia A *vs* Itatiaia B. Click here for file

Additional file 6: Table S6 Summarized features of the marginal histograms for each parameter to the pairwise comparison Florianópolis *vs* Itatiaia A (A) and Florianópolis *vs* Itatiaia B (B). Click here for file
