# Supplementary material for: Evidence for the occurrence of two sympatric sibling species within the Anopheles (Kerteszia) cruzii complex in southeast Brazil and the detection of asymmetric introgression between them using a multilocus analysis
Source: BMC Evol Biol. 2013 Sep 24;13:207. doi: 10.1186/1471-2148-13-207 (PMC3850420; doi:10.1186/1471-2148-13-207)
Supplement: Additional file 1: Table S1 — NR blocks and sequences excluded from the IM analysis. Edition of sequences prior to IM analysis using the IMGC program and based on alignments presented in Additional file 3: Table S3. NR blocks, fragment positions of the non-recombining blocks used in the analyses; Removed sequences, the putative recombinant sequences removed before the IM analysis. [file 1471-2148-13-207-S1.pdf]

locus	NR blocks	Removed sequences
timeless	236 – 361	Flo30a, Flo30b, Flo31a, Flo34a, Flo34b, Ita01a
Clock	035 – 195	Ita05b, Ita06b, Ita09a
cycle	061 – 131	Flo06a, Flo09b, Ita04b, Ita07a, Ita08a, Ita09b, Ita12a, Ita12b
Rp49	049 – 269	Flo06a, Flo06b, Flo09b, Flo12b, Ita05b
RpS2	001 – 214	Flo03b, Flo09b, Flo11b, Ita13b
RpS29	052 – 191	Flo09b, Flo12a, Ita04a, Ita05a, Ita06a, Ita06b, Ita07a, Ita08b, Ita13a, Ita13b
