# Supplementary material for: Evidence for the occurrence of two sympatric sibling species within the Anopheles (Kerteszia) cruzii complex in southeast Brazil and the detection of asymmetric introgression between them using a multilocus analysis
Source: BMC Evol Biol. 2013 Sep 24;13:207. doi: 10.1186/1471-2148-13-207 (PMC3850420; doi:10.1186/1471-2148-13-207)
Supplement: Additional file 2: Table S2 — Polymorphism summaries of An. cruzii sibling species. RM, the minimum number of recombination events; n, number of DNA sequences of each sibling species; S, number of polymorphic (segregating) sites; θ, nucleotide diversity based on the total number of mutations (Eta); π, nucleotide diversity based on the average number of pair-wise differences; DT, Tajima’s D [28]; DFL, Fu & Li’s D [29] and FFL, Fu & Li’s F [29], based on Eta (total number of mutations). No significant deviations from neutrality were observed after Bonferroni correction. Numbers in parentheses are related to the non-recombining block (NR) for each locus. [file 1471-2148-13-207-S2.pdf]

Locus	Population	*RM*	*Length (bp)*	*n*	S	θ	π	D_T	D_{FL}	F_{FL}
timeless	Florianópolis	13	414 (126)	24 (19)	59 (21)	0.0411 (0.0449)	0.0308 (0.0306)	-0.9832 (-1.1685)	-0.5497 (-0.8613)	-0.8051 (-1.1169)
	Itatiaia			24 (23)	29 (05)	0.0198 (0.0112)	0.0189 (0.0083)	-0.1858 (-0.7414)	-0.5269 (-1.3380)	-0.4935 (-1.3515)
	Itatiaia A			12 (12)	10 (05)	0.0081 (0.0095)	0.0111 (0.0120)	1.5331 (1.0364)	1.0363 (0.5626)	1.3229 (0.7739)
	Itatiaia B			08 (08)	17 (04)	0.0168 (0.0086)	0.0171 (0.0065)	0.0697 (-1.0297)	0.1856 (-0.9208)	0.1763 (-1.0399)
Clock	Florianópolis	02	195 (161)	24 (24)	10 (10)	0.0171 (0.0217)	0.0177 (0.0225)	0.1124 (0.1124)	-0.6073 (-0.6073)	-0.4584 (-0.4584)
	Itatiaia			24 (21)	15 (14)	0.0341 (0.0419)	0.0250 (0.0311)	-0.9617 (-0.9283)	0.6915 (0.6362)	0.2194 (0.1874)
	Itatiaia A			12 (12)	07 (07)	0.0157 (0.0157)	0.0128 (0.0128)	-0.6647 (-0.6647)	-0.2517 (-0.2517)	-0.4057 (-0.4057)
	Itatiaia B			08 (07)	13 (14)	0.0405 (0.0462)	0.0400 (0.0428)	0.1584 (-0.4094)	-0.3159 (0.0189)	-0.4265 (-0.0849)
cycle	Florianópolis	08	218 (71)	24 (22)	21 (12)	0.0270 (0.0490)	0.0280 (0.0377)	0.1357 (-0.8009)	0.5674 (-0.1505)	0.5089 (-0.4032)
	Itatiaia			24 (18)	18 (08)	0.0233 (0.0301)	0.0231 (0.0297)	-0.0317 (-0.0503)	-0.2068 (0.1404)	-0.1794 (0.0981)
	Itatiaia A			12 (11)	10 (05)	0.0154 (0.0134)	0.0187 (0.0163)	0.9496 (0.9022)	0.2429 (0.5974)	0.4833 (0.7594)
	Itatiaia B			08 (08)	12 (09)	0.0217 (0.0277)	0.0235 (0.0312)	0.5596 (0.7968)	0.6619 (0.7366)	0.7078 (0.8311)
Rp49	Florianópolis	02	271 (221)	24 (20)	10 (09)	0.0111 (0.0123)	0.0084 (0.0089)	-0.8207 (-0.9168)	-0.4332 (-0.6073)	-0.6392 (-0.8139)
	Itatiaia			22 (21)	16 (12)	0.0163 (0.0149)	0.0150 (0.0107)	-0.2790 (-0.9741)	0.5352 (0.1902)	0.3410 (-0.1775)
	Itatiaia A			10 (10)	05 (05)	0.0066 (0.0066)	0.0074 (0.0074)	0.5276 (0.5276)	-0.0239 (-0.0239)	0.1245 (0.1245)
	Itatiaia B			08 (07)	08 (08)	0.0116 (0.0121)	0.0134 (0.0127)	0.8177 (0.2626)	1.0497 (0.3218)	1.1015 (0.3374)
RpS2	Florianópolis	02	267 (214)	24 (21)	17 (14)	0.0183 (0.0187)	0.0172 (0.0168)	-0.2287 (-0.3654)	0.9497 (0.7993)	0.6908 (0.5223)
	Itatiaia			24 (23)	14 (11)	0.0140 (0.0137)	0.0126 (0.0126)	-0.3410 (-0.2749)	0.7378 (0.5022)	0.4817 (0.3152)
	Itatiaia A			12 (12)	09 (08)	0.0113 (0.0123)	0.0116 (0.0126)	0.1713 (0.0721)	0.1089 (-0.0526)	0.1419 (-0.0233)
	Itatiaia B			08 (08)	04 (03)	0.0058 (0.0054)	0.0049 (0.0035)	-0.6257 (-1.4475)	-0.9208 (-1.5653)	-0.9372 (-1.6858)
RpS29	Florianópolis	03	274 (140)	24 (22)	13 (11)	0.0135 (0.0239)	0.0090 (0.0140)	-1.1475 (-1.4039)	-2.1989 (-2.3043)	-2.1961 (-2.3716)
	Itatiaia			24 (16)	25 (16)	0.0257 (0.0340)	0.0210 (0.0251)	-0.6798 (-0.9320)	-0.9492 (-1.2205)	-1.0138 (-1.32309)
	Itatiaia A			12 (10)	12 (05)	0.0147 (0.0094)	0.0160 (0.0122)	0.4282 (1.2838)	0.1135 (-0.1537)	0.2217 (-0.1104)
	Itatiaia B			08 (08)	15 (12)	0.0228 (0.0269)	0.0185 (0.0213)	-0.8523 (-0.9158)	-1.0337 (-1.0729)	-1.1013 (-1.1496)
