# Supplementary material for: Evidence for the occurrence of two sympatric sibling species within the Anopheles (Kerteszia) cruzii complex in southeast Brazil and the detection of asymmetric introgression between them using a multilocus analysis
Source: BMC Evol Biol. 2013 Sep 24;13:207. doi: 10.1186/1471-2148-13-207 (PMC3850420; doi:10.1186/1471-2148-13-207)

Alignment of the DNA sequences from the *timeless* gene fragment from Florianópolis and Itatiaia.

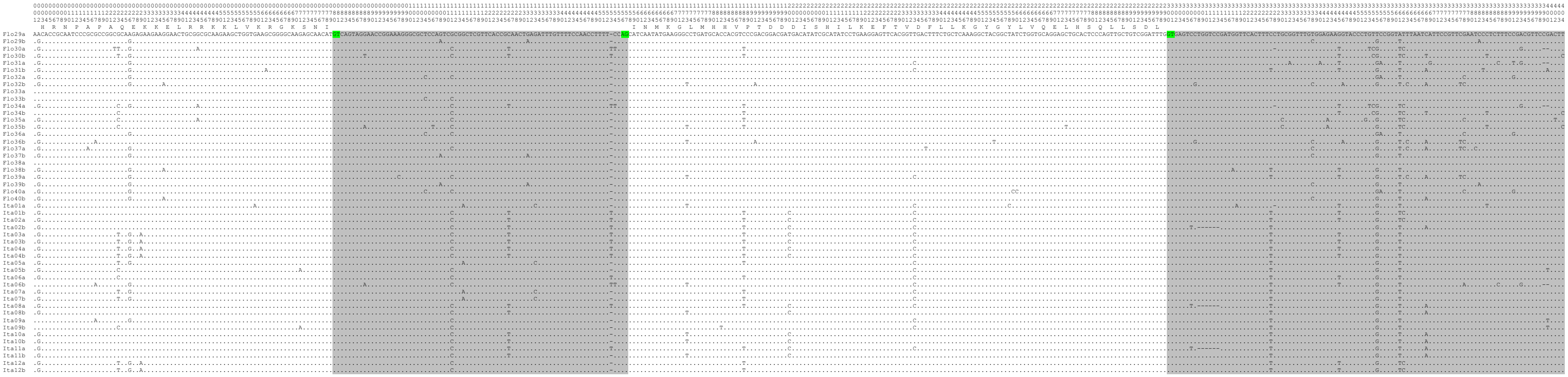

Alignment of the DNA sequences from the *Clock* gene fragment from Florianópolis and Itatiaia.

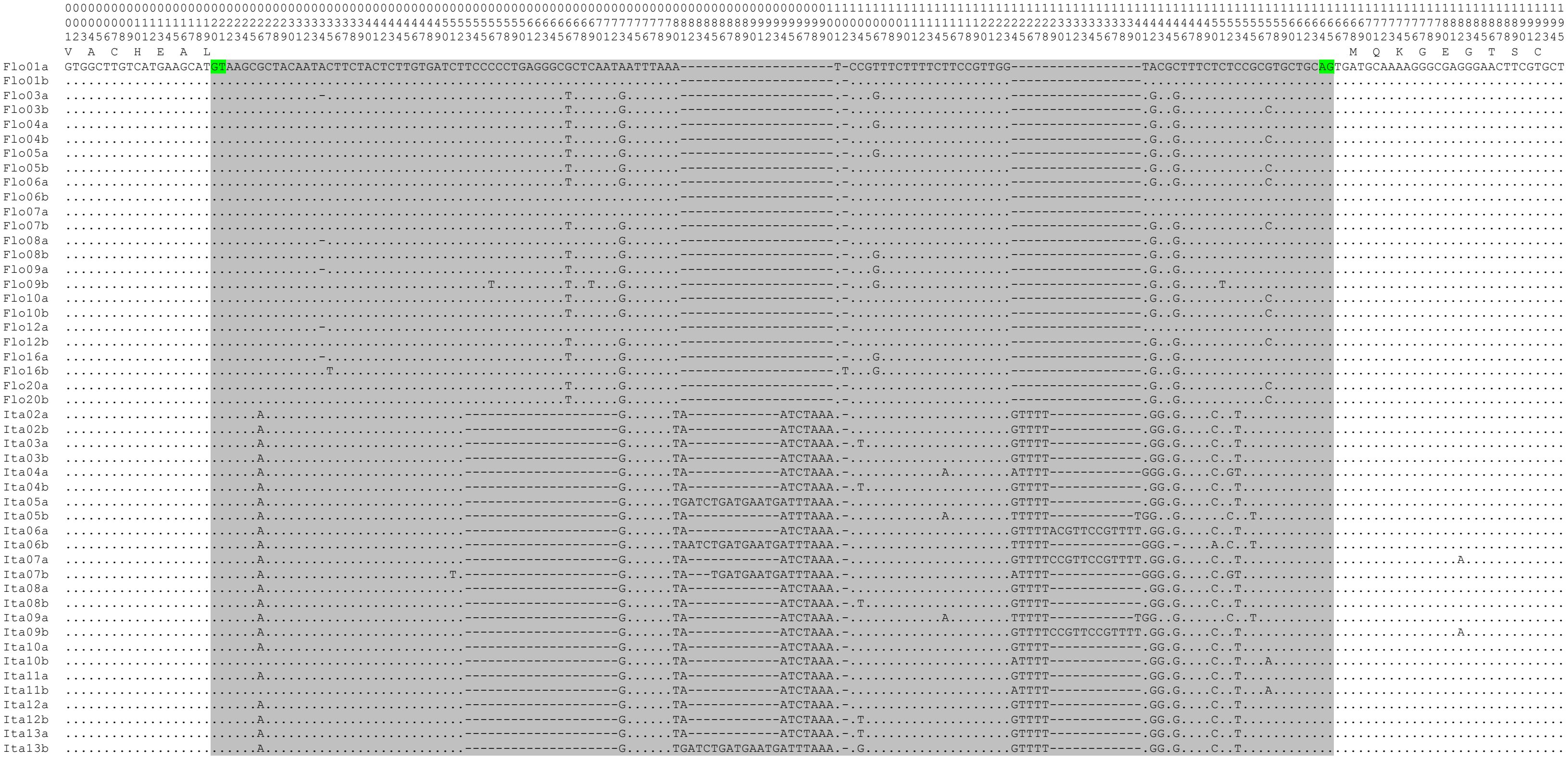

Alignment of the DNA sequences from the *cycle* gene fragment from Florianópolis and Itatiaia.

```
       000000000000000000000000000000000000000000000000000000000000000000000000000000000000000000000000000111111111111111111111111111111111111111111111111111111111111111111111111111111111111111111111111111112222222222222222222
       000000000111111111122222222223333333333444444444455555555556666666666777777777788888888889999999999000000000011111111112222222222333333333344444444445555555555666666666677777777778888888888999999999900000000001111111111
       123456789012345678901234567890123456789012345678901234567890123456789012345678901234567890123456789012345678901234567890123456789012345678901234567890123456789012345678901234567890123456789012345678901234567890123456789012345678
       S  A  M  I  P  M  C  H  A  M  S  R  K  L  D  K  L  T  V  L  R  M  A  V  Q  H  L  K  T  I  R  G  A  V  H  S  Y  T  E  G  H  Y  K  P  A  F  L  S  D  Q  E  L  K  M  L  I  L  Q  A  A  E  G  F  L  F  V  V  G  C  D  R  G
Ita08b TCCGCGATGATACCGATGTGCCACGCGATGTCCCGAAAGCTGGACAAGCTGACGGTGCTGCGGATGGCGGTGCAGCACCTGAAGACGATCCGCGGCGCAGTCCACTCGTACACGGAGGGCCACTACAAACCAGCCTTTCTGTCCGACCAGGAGCTGAAGATGCTGATCCTGCAGGCGGCCGAAGGCTTCCTGTTCGTGGTCGGGTGCGATCGGGGCCG
Ita02a ..........................................................................................................................................................................................................................
Ita02b ....................T.....A.........................................C...............................................A...........G...............................................A.........................................
Ita03a ..........................A.....................................................................................................G..........................C..............................................................
Ita03b ................................................................................................................................G..........................C..............................................................
Ita04a ..........................A.....................................................................................................G..........................C..............................................................
Ita04b ....................T.....A.........................................................................................A..T........G.........................................................................................
Ita05a ....................................................................C.......................T........T..........................G..G.......................C...............................................A..............
Ita05b ....................................................................C.......................T........T..........................G..G.......................C...............................................A..............
Ita06a ....................................................................C...............................................A...........G...............................................A.........................................
Ita06b ....................................................................C...............................................A...........G...............................................A.........................................
Ita07a ..........................A.........................................................................................A...........G.................................T.............A.........................................
Ita07b .......................................T............................C...........................................................G..G.......................C................................T.............................
Ita08a ....................................................................C....................T.................C...............................................C..............................................................
Ita09a ................................................................................................................................G..G.......................C..............................................................
Ita09b ..........................A.........................................C.......................T...................................G..G.......................C..............................................................
Ita10a ....................T.....A.........................................C...............................................A...........G...............................................A.........................................
Ita10b ..........................................................................................................................................................................................................................
Ita11a ....................T.....A.........................................C...............................................A...........G...............................................A.........................................
Ita11b ....................T.....A.........................................C...............................................A...........G...............................................A.........................................
Ita12a ..........................A.........................................................................................A..T........G..T......................................................................................
Ita12b ..........................A.........................................................................................A..T........G..T......................................................................................
Ita13a ....................................................................C...........................................................G..G...................................T...................................A..............
Ita13b ....................T.....A.........................................C...............................................A...........G...............................................A.........................................
Flo01a ..............................................................A.....C........................................................T..G..........................C.................A.........................................T..
Flo01b ..............................................................A.....C........................................................T..G..........................C.................A.........................................T..
Flo03a ....................................................................C................................T..........................G..........................C................................T..............T...........T..
Flo03b ....................................................................C................................T..........................G..........................C................................T..............T...........T..
Flo05a ....................................................................C................................T..........................G..........................C................................T..............T...........T..
Flo05b ....................................................................C................................T..........................G..........................C................................T..............T...........T..
Flo06a ..........................A...........................................................A.....T........T..............A..............G........................................................T..........................T..
Flo06b ..........................A.............................A...........C.......................T........T..........................G..G...........G.......................................................................T..
Flo07a ........................................................A...........C....................T......................................G..........................C................................T..............T...........T..
Flo07b ........................................................A...........C....................T......................................G..........................C................................T..........................T..
Flo08a ....................................................................T..........................................T.................G..G...........G.......................................................................T..
Flo08b ........................................................A...........C....................T......................................G..........................C................................T..........................T..
Flo09a ........................................................A...........C....................T......................................G..........................C................................T..............T...........T..
Flo09b ............................................................................................T........T..........................G..G.......................C..............................................................
Flo11a ........................................................A...A.......C....................T......................................G..........................C................................T..........................T..
Flo11b ....................................................................C................................T..........................G..G...........G............................................T..........................T..
Flo12a ........................................................A...........C....................T......................................G..........................C................................T..............T...........T..
Flo12b ....................................................................C................................T..........................G..G...........G..........................................................................
Flo13a ....................................................................T..........................................T.................G..G...........G.......................................................................T..
Flo13b ....................................................................C................................T..........................G..G...........G..........................................................................
Flo17a ....................................................................C................................T..........................G..........................C................................T..............T...........T..
Flo17b ....................................................................C................................T..........................G..........................C................................T..............T...........T..
Flo18a ..............................................................A.....C..........................T.............................T..G..........................C.................A.........................................T..
Flo18b ..............................................................A.....C..........................T.............................T..G..........................C.................A.........................................T..
```

Alignment of the DNA sequences from the *Rp49* gene fragment from Florianópolis and Itatiaia.

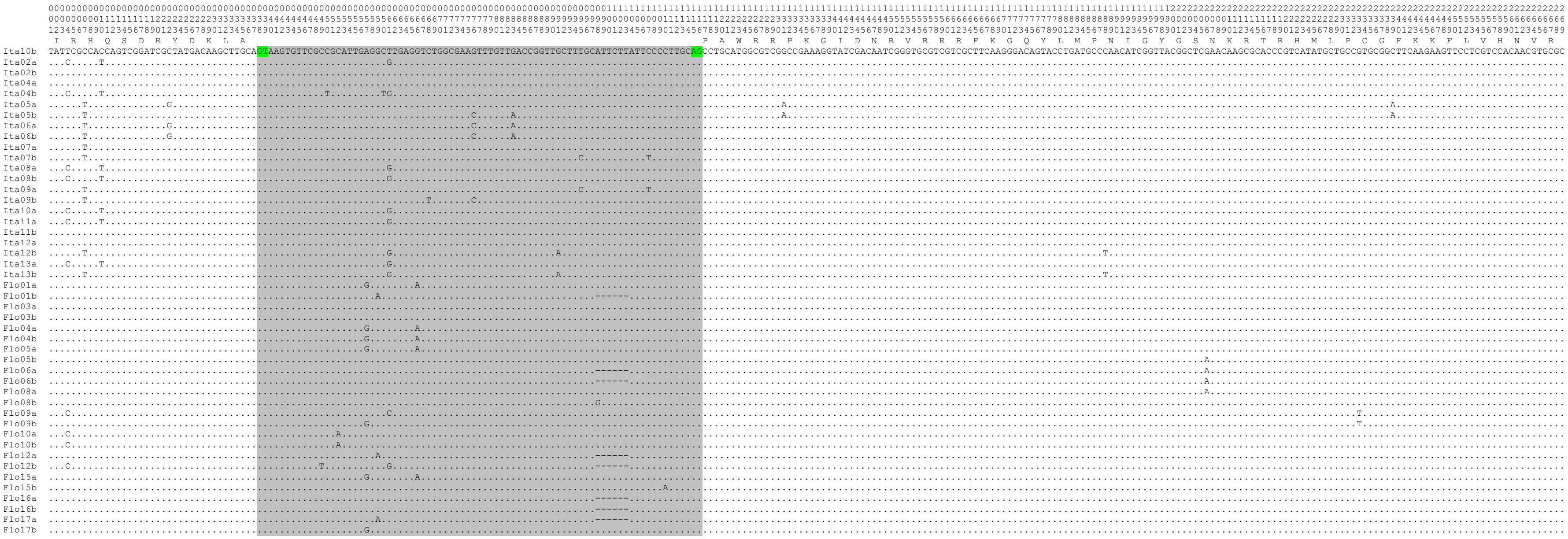

Alignment of the DNA sequences from the *RpS2* gene fragment from Florianópolis and Itatiaia.

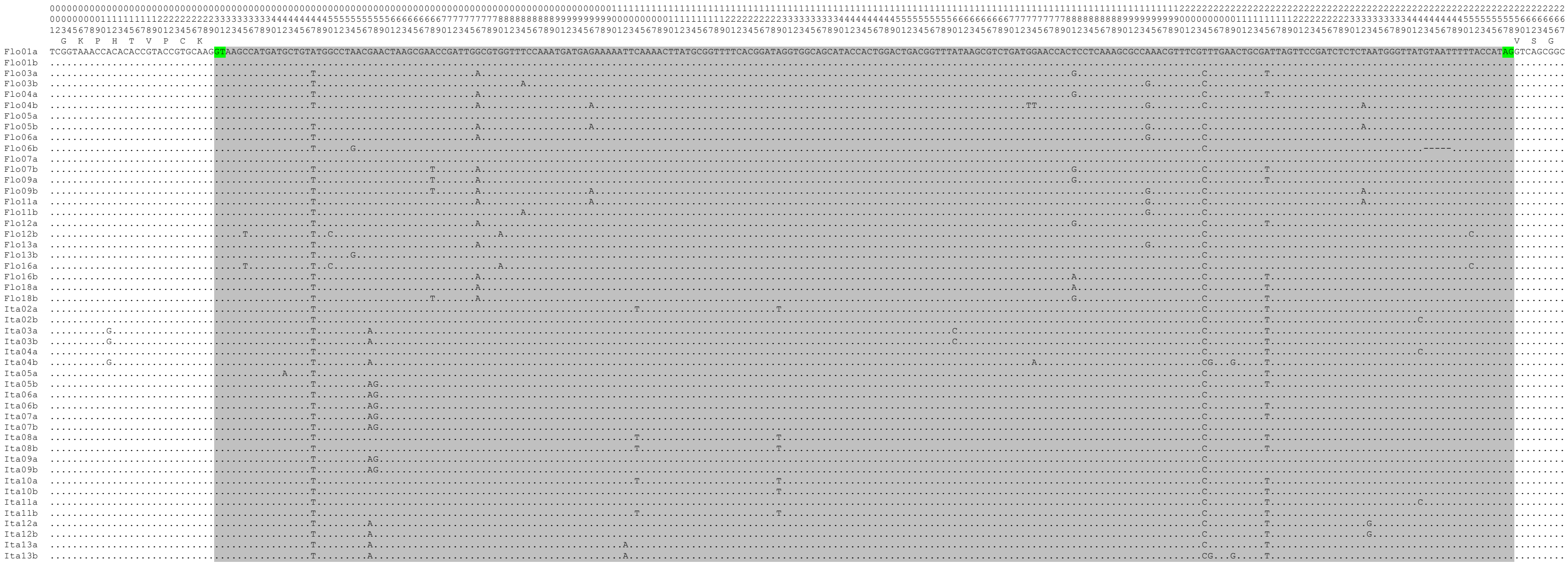

Alignment of the DNA sequences from the *RpS29* gene fragment from Florianópolis and Itatiaia.

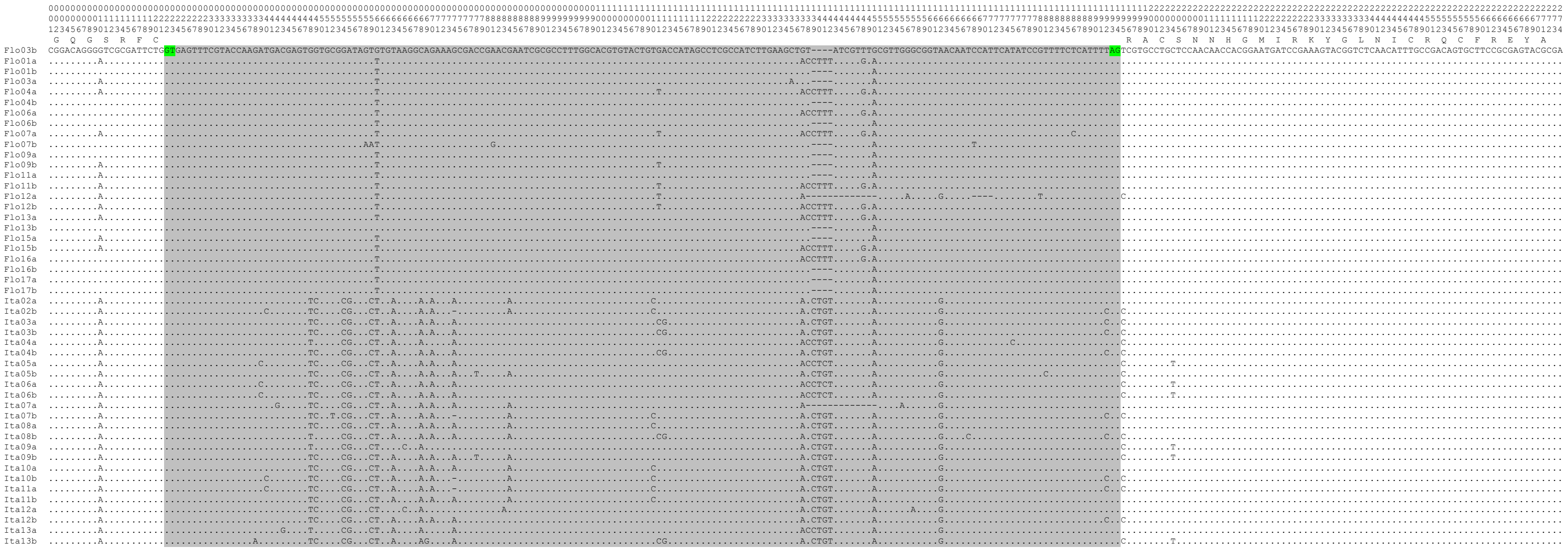

Supplement: Additional file 3: Table S3 — Alignments of the DNA sequences of the timeless, Clock, cycle, Rp49, RpS2 and RpS29 gene fragments from Florianópolis and Itatiaia. The translated amino acid sequences are shown above the alignments and the introns are highlighted in grey. Dots represent identity and dashed represent gaps. Flo: individuals from Florianópolis; Ita: individuals from Itatiaia. [file 1471-2148-13-207-S3.pdf]
