# Supplementary material for: Evidence for the occurrence of two sympatric sibling species within the Anopheles (Kerteszia) cruzii complex in southeast Brazil and the detection of asymmetric introgression between them using a multilocus analysis
Source: BMC Evol Biol. 2013 Sep 24;13:207. doi: 10.1186/1471-2148-13-207 (PMC3850420; doi:10.1186/1471-2148-13-207)
Supplement: Additional file 4: Table S4 — Summarized features of the marginal histograms for each parameter for the pairwise comparison Florianópolis vs Itatiaia. [file 1471-2148-13-207-S4.pdf]

		Minbin	Maxbin	HiPt	HiSmth	Mean	95Lo	95Hi	HPD90Lo	HPD90Hi
θ_1	**A**	1.2916	8.5312	3.2095	3.2699	3.3563	2.2592	4.9287	2.3110	4.4967
	**B**	1.0324	8.6349	3.2613	3.2181	3.3563	2.2505	4.9287	2.2937	4.4881
	**C**	1.0929	8.6263	3.2181	3.2095	3.3477	2.2419	4.9200	2.2937	4.4881
	**D**	1.1274	8.6090	3.2008	3.1922	3.3477	2.2505	4.9200	2.2937	4.4794
θ_2	**A**	0.7779	6.4783	2.1300	2.1232	2.2795	1.4710	3.4956	1.4913	3.1355
	**B**	0.6217	6.7908	2.2047	2.1843	2.2795	1.4710	3.4956	1.4981	3.1423
	**C**	0.6013	6.7840	2.1775	2.1708	2.2795	1.4710	3.4956	1.4981	3.1423
	**D**	0.6488	6.7772	2.1640	2.1572	2.2795	1.4710	3.5024	1.4913	3.1355
θ_A	**A**	0.0043	8.6349	0.7473	0.7041	0.8510	0.0475	2.7257	0.0043	1.8877
	**B**	0.0043	8.6349	0.6782	0.6609	0.8423	0.0475	2.7170	0.0043	1.8790
	**C**	0.0043	8.6349	0.0043	0.0043	0.8423	0.0475	2.7170	0.0043	1.8704
	**D**	0.0043	8.6349	0.6523	0.6436	0.8510	0.0475	2.7343	0.0043	1.8877
t	**A**	0.5740	3.9980	1.4740	1.5020	1.5900	1.0180	2.3980	1.0420	2.1660
	**B**	0.4500	3.9980	1.5260	1.5500	1.5980	1.0260	2.4100	1.0500	2.1820
	**C**	0.3700	3.9980	1.5380	1.5340	1.6020	1.0260	2.4140	1.0500	2.1780
	**D**	0.5020	3.9980	1.5060	1.5260	1.5940	1.0220	2.4100	1.0460	2.1780
m_1	**A**	0.0010	1.1630	0.0430	0.0470	0.0830	0.0110	0.3010	0.0030	0.2030
	**B**	0.0010	1.4170	0.0490	0.0470	0.0870	0.0110	0.3090	0.0030	0.2110
	**C**	0.0010	1.3030	0.0470	0.0470	0.0870	0.0110	0.3110	0.0030	0.2110
	**D**	0.0010	1.2710	0.0470	0.0470	0.0870	0.0110	0.3110	0.0030	0.2110
m_2	**A**	0.0010	0.9210	0.0350	0.0330	0.0690	0.0050	0.2650	0.0010	0.1770
	**B**	0.0010	1.3630	0.0310	0.0330	0.0670	0.0050	0.2630	0.0010	0.1730
	**C**	0.0010	1.6010	0.0330	0.0330	0.0670	0.0050	0.2630	0.0010	0.1730
	**D**	0.0010	1.1210	0.0310	0.0310	0.0670	0.0050	0.2630	0.0010	0.1730
