# Supplementary material for: Evidence for the occurrence of two sympatric sibling species within the Anopheles (Kerteszia) cruzii complex in southeast Brazil and the detection of asymmetric introgression between them using a multilocus analysis
Source: BMC Evol Biol. 2013 Sep 24;13:207. doi: 10.1186/1471-2148-13-207 (PMC3850420; doi:10.1186/1471-2148-13-207)
Supplement: Additional file 5: Table S5 — Summarized features of the marginal histograms for each parameter for the pairwise comparison Itatiaia A vs Itatiaia B. [file 1471-2148-13-207-S5.pdf]

		Minbin	Maxbin	HiPt	HiSmth	Mean	95Lo	95Hi	HPD90Lo	HPD90Hi
θ_1	**A**	0.0557	4.1738	0.7394	0.6917	0.7394	0.2306	1.5026	0.1988	1.2482
	**B**	0.0398	4.5872	0.6917	0.7076	0.7394	0.2306	1.5026	0.1988	1.2482
	**C**	0.0557	4.1102	0.7076	0.6917	0.7235	0.2306	1.5026	0.1988	1.2482
	**D**	0.0398	6.6701	0.7076	0.6917	0.7394	0.2306	1.5026	0.1988	1.2482
θ_2	**A**	0.1095	24.3182	1.3017	1.2530	1.5450	0.6204	4.7323	0.4501	3.0048
	**B**	0.1095	24.3182	1.2530	1.2530	1.5450	0.6204	4.6106	0.4501	2.9805
	**C**	0.1581	24.3182	1.3260	1.2530	1.5450	0.6204	4.6593	0.4501	3.0048
	**D**	0.0852	24.3182	1.2287	1.2530	1.5450	0.6204	4.6349	0.4501	2.9805
m_1	**A**	0.0050	9.9950	0.0050	0.0050	0.7750	0.0250	7.6150	0.0050	4.3950
	**B**	0.0050	9.9950	0.0050	0.0050	0.7550	0.0150	7.5550	0.0050	4.2950
	**C**	0.0050	9.9950	0.0050	0.0050	0.7950	0.0250	7.6350	0.0050	4.4450
	**D**	0.0050	9.9950	0.0050	0.0050	0.7750	0.0250	7.6350	0.0050	4.4050
m_2	**A**	0.0050	9.9950	2.2050	2.1950	2.4950	0.2950	6.2450	0.0050	4.5150
	**B**	0.0050	9.9950	2.1950	2.2050	2.5050	0.3150	6.2450	0.0750	4.5850
	**C**	0.0050	9.9950	2.2550	2.2450	2.4950	0.3050	6.2150	0.0750	4.5650
	**D**	0.0050	9.9950	2.2350	2.2250	2.5050	0.2950	6.2350	0.0250	4.5350
