# Supplementary material for: Evidence for the occurrence of two sympatric sibling species within the Anopheles (Kerteszia) cruzii complex in southeast Brazil and the detection of asymmetric introgression between them using a multilocus analysis
Source: BMC Evol Biol. 2013 Sep 24;13:207. doi: 10.1186/1471-2148-13-207 (PMC3850420; doi:10.1186/1471-2148-13-207)
Supplement: Additional file 6: Table S6 — Summarized features of the marginal histograms for each parameter to the pairwise comparison Florianópolis vs Itatiaia A (A) and Florianópolis vs Itatiaia B (B). [file 1471-2148-13-207-S6.pdf]

A

		Minbin	Maxbin	HiPt	HiSmth	Mean	95Lo	95Hi	HPD90Lo	HPD90Hi
θ_1	**A**	1.1533	8.6003	3.2267	3.2181	3.3218	2.1987	4.9114	2.2505	4.4622
	**B**	0.9460	8.6263	3.1749	3.1663	3.3131	2.1987	4.9114	2.2419	4.4622
	**C**	1.0583	8.5917	3.1404	3.2008	3.3131	2.1987	4.9114	2.2419	4.4622
	**D**	1.0151	8.6349	3.2181	3.2095	3.3218	2.1987	4.9114	2.2505	4.4708
θ_2	**A**	0.2274	4.2191	1.0394	1.0351	1.1031	0.6100	1.9491	0.6058	1.6728
	**B**	0.1892	4.2488	1.0266	1.0181	1.1031	0.6100	1.9448	0.6015	1.6643
	**C**	0.2104	4.2488	1.0309	1.0266	1.1031	0.6100	1.9448	0.6058	1.6685
	**D**	0.1934	4.2488	1.0139	1.0096	1.1031	0.6100	1.9448	0.6058	1.6685
θ_A	**A**	0.0043	8.6349	0.8855	0.8769	1.1015	0.0734	3.5464	0.0043	2.3542
	**B**	0.0043	8.6349	0.9114	0.9028	1.1015	0.0734	3.5464	0.0043	2.3628
	**C**	0.0043	8.6349	0.8769	0.8769	1.1101	0.0734	3.5464	0.0043	2.3628
	**D**	0.0043	8.6349	0.8596	0.9201	1.1015	0.0734	3.5291	0.0043	2.3542
t	**A**	0.3500	3.9980	1.3700	1.3660	1.4540	0.8740	2.5460	0.8700	2.1100
	**B**	0.3380	3.9980	1.3300	1.3540	1.4540	0.8780	2.5580	0.8700	2.1100
	**C**	0.3980	3.9980	1.3500	1.3460	1.4500	0.8820	2.5500	0.8700	2.1020
	**D**	0.3140	3.9980	1.3660	1.3620	1.4580	0.8780	2.5340	0.8740	2.1100
m_1	**A**	0.0010	1.2290	0.0450	0.0450	0.0790	0.0110	0.2890	0.0030	0.1950
	**B**	0.0010	1.5030	0.0470	0.0450	0.0790	0.0110	0.2930	0.0030	0.1950
	**C**	0.0010	1.5670	0.0410	0.0450	0.0790	0.0090	0.2930	0.0030	0.1950
	**D**	0.0010	1.3550	0.0470	0.0450	0.0790	0.0110	0.2910	0.0030	0.1950
m_2	**A**	0.0010	1.9970	0.0010	0.0010	0.0670	0.0030	0.4150	0.0010	0.2410
	**B**	0.0010	1.9990	0.0010	0.0010	0.0690	0.0030	0.4170	0.0010	0.2410
	**C**	0.0010	1.9950	0.0010	0.0010	0.0690	0.0030	0.4170	0.0010	0.2410
	**D**	0.0010	1.9990	0.0010	0.0010	0.0670	0.0030	0.4150	0.0010	0.2410

B

		Minbin	Maxbin	HiPt	HiSmth	Mean	95Lo	95Hi	HPD90Lo	HPD90Hi
θ_1	**A**	1.1895	8.8523	3.3102	3.3013	3.4527	2.3033	5.0833	2.3568	4.6378
	**B**	1.0737	8.9058	3.3458	3.3369	3.4616	2.3122	5.0922	2.3568	4.6378
	**C**	1.1895	8.8523	3.2567	3.3547	3.4616	2.3122	5.0833	2.3568	4.6289
	**D**	1.1361	8.9058	3.3458	3.3102	3.4616	2.3122	5.0922	2.3568	4.6378
θ_2	**A**	0.3221	7.0766	1.7453	1.7382	1.9293	1.0018	3.7419	0.9594	3.0834
	**B**	0.2230	7.0766	1.7311	1.7240	1.9293	0.9948	3.7419	0.9594	3.0834
	**C**	0.3292	7.0766	1.7169	1.6886	1.9364	1.0018	3.7348	0.9664	3.0905
	**D**	0.2230	7.0766	1.6957	1.7453	1.9293	0.9948	3.7348	0.9594	3.0834
θ_A	**A**	0.0045	8.9058	0.0312	0.0401	0.7173	0.0312	2.8201	0.0045	1.8311
	**B**	0.0045	8.9058	0.0045	0.0045	0.7173	0.0312	2.8201	0.0045	1.8400
	**C**	0.0045	8.9058	0.0936	0.0846	0.7173	0.0401	2.8201	0.0045	1.8400
	**D**	0.0045	8.9058	0.0045	0.0045	0.7173	0.0312	2.8201	0.0045	1.8400
t	**A**	0.4780	3.9980	1.6180	1.6620	1.7060	1.0860	2.5980	1.1140	2.3140
	**B**	0.4300	3.9980	1.6340	1.6660	1.7020	1.0780	2.5860	1.1100	2.3100
	**C**	0.4100	3.9980	1.6980	1.6780	1.7020	1.0740	2.5780	1.1100	2.3100
	**D**	0.3660	3.9980	1.6780	1.6740	1.7020	1.0780	2.5820	1.1100	2.3100
m_1	**A**	0.0010	1.9830	0.0530	0.0510	0.0970	0.0110	0.3430	0.0010	0.2310
	**B**	0.0010	1.6070	0.0530	0.0530	0.0950	0.0090	0.3370	0.0010	0.2270
	**C**	0.0010	1.5530	0.0590	0.0570	0.0950	0.0110	0.3370	0.0010	0.2270
	**D**	0.0010	1.7770	0.0530	0.0510	0.0950	0.0090	0.3370	0.0010	0.2270
m_2	**A**	0.0010	1.9990	0.0470	0.0450	0.1530	0.0090	0.6690	0.0010	0.4290
	**B**	0.0010	1.9990	0.0510	0.0490	0.1530	0.0090	0.6730	0.0010	0.4290
	**C**	0.0010	1.9990	0.0470	0.0550	0.1530	0.0090	0.6650	0.0010	0.4250
	**D**	0.0010	1.9990	0.0510	0.0530	0.1530	0.0090	0.6730	0.0010	0.4310
